# All-cause mortality and overdose deaths among 4192 people who inject drugs in Stockholm: a 10-year register-based cohort study

**DOI:** 10.1186/s12954-026-01407-z

**Published:** 2026-02-09

**Authors:** E. Holmén, A. Hammarberg, M. Kåberg

**Affiliations:** 1 Centre for Psychiatry Research, Department of Clinical Neuroscience, Karolinska Institutet, and Stockholm Health Care Services, Region Stockholm, Tomtebodavägen 18A, 17176 Solna, Sweden; 2 Stockholm Centre for Dependency Disorders, Stockholm Health Care Services, Region Stockholm, Stockholm, Sweden; 3 Department of Global Public Health, Karolinska Institutet, Stockholm, Sweden; 4 Stockholm Centre for Dependency Disorders, Stockholm Needle and Syringe Program, Stockholm, Sweden

**Keywords:** People who inject drugs, All-cause mortality, Overdose mortality, Needle and syringe programs, Take-Home naloxone, Sweden

## Abstract

**Background:**

People who inject drugs (PWID) face premature mortality, particularly from opioid overdose. In Sweden, harm reduction has expanded, including increased access to opioid agonist therapy (OAT) and the 2018 introduction of Take-Home Naloxone (THN). This study aimed to examine trends in all-cause and cause-specific mortality and to estimate predictors of all-cause and opioid overdose mortality among PWID in Stockholm.

**Methods:**

We conducted a retrospective cohort study from April 2013 to March 2023. Data from the national Cause of Death Register were linked to Stockholm Needle and Syringe Program (NSP) records. Causes of death were categorised as opioid overdoses, external causes, internal/natural causes, or other/unknown. Crude mortality rates and age- and sex-standardised mortality ratios were calculated. Time-dependent Cox regression models estimated risk of all-cause death, and Fine and Gray subdistribution hazard models estimated opioid overdose mortality, accounting for competing risks. Person-time began at first NSP visit and ended at death, study end or censoring (> 365 days without a visit).

**Results:**

Among 4192 participants, 685 (16%) died. The crude mortality rate declined from 36.75 to 27.04 deaths per 1,000 person-years and the standardised mortality ratio from 17.24 to 10.94. In multivariable models, reporting opioids as the latest injected drug was the strongest driver for both all-cause and opioid mortality. Other significant predictors included infrequent injecting and current contact with social services, psychiatry or addiction care, while OAT participation was associated with a lower risk. Male sex and age over 56 at enrolment were associated with a higher risk of all-cause mortality. Opioid overdose was the most common cause of death (53%). However, the opioid overdose mortality rate declined from 29.40 to 5.88 deaths per 1,000 person-years over the study period, coinciding with the 2018 introduction of THN and declining reported opioid injecting drug use among NSP clients.

**Conclusions:**

All-cause mortality among PWID in Stockholm declined over the study period, alongside significant reductions in opioid overdose deaths, during a period of broadened harm reduction and reduced reporting of opioid injecting drug use. Our findings support continued scale-up of OAT and THN and consideration of supervised consumption sites to further reduce preventable deaths.

**Supplementary Information:**

The online version contains supplementary material available at 10.1186/s12954-026-01407-z.

## Background

There are, worldwide, an estimated 14.8 million people who inject drugs (PWID), and they experience markedly reduced life expectancies compared to the general population [1, 2]. Accidental opioid overdose is the leading cause of death, but other factors, including human immunodeficiency virus (HIV) [3, 4], suicide [5], trauma [6], and cardiovascular diseases [7], also significantly contribute to premature mortality.

The elevated mortality among PWID reflects a complex interplay of individual behaviours and broader social determinants. Beyond the direct physical risks of drug use, factors such as homelessness [8, 9], socioeconomic marginalisation [10], and unsafe injection practices [11] further increase the risk of premature death. Additionally, critical periods such as release from prison or institutional care are associated with an especially high risk of fatal overdose, partly due to reduced drug tolerance following a period of enforced abstinence [12, 13].

In Sweden, where an estimated 21,000 individuals inject drugs [14], drug-related mortality remains high compared to most European countries [15]. Swedish studies on mortality in populations using drugs largely align with global findings, identifying overdose, suicide, trauma and chronic diseases as common causes of death [7, 16–22], with risk factors for all-cause mortality including male sex, older age, HIV infection and opioid use [7, 16, 18, 22]. However, most national mortality studies focus on individuals in treatment settings with various modes of intake, with limited research exclusively on PWID.

In response to the high mortality rates among PWID, harm reduction strategies, including needle and syringe programs (NSP), supervised consumption sites (SCS), opioid agonist therapy (OAT), and Take-Home Naloxone (THN) programs, have been implemented worldwide, aiming to reduce blood-borne diseases and overdose deaths [23–27]. Furthermore, advances in treatment for HIV and hepatitis C have improved health outcomes for PWID and may contribute to reduced mortality [4, 28].

Although Swedish drug policy is characterised by a strict zero-tolerance approach (i.e. no acceptance of drug use or possession), harm reduction services have expanded over the past decade [29, 30]. Notably, the Stockholm NSP pioneered Sweden’s first THN program in 2018, contributing to a substantial number of overdose reversals [31]. Preliminary evidence suggests that such programs may contribute to reduced overdose mortality. For example in southern Sweden, a large-scale regional THN initiative was associated with lower rates of overdose deaths in the three years following implementation [32].

Drawing on a large cohort and high-quality linked data, the objective of our study was to examine trends in all-cause and cause-specific mortality among PWID registered at the Stockholm NSP over a 10-year period. Additionally, we aimed to estimate predictors for all-cause and opioid overdose mortality, leveraging comprehensive time-dependent data on demographics, drug use patterns, and social determinants.

## Methods

### Design and study setting

This was a retrospective cohort study using data from two different registers (described in detail below) to assess mortality and causes of death among PWID in Stockholm during the study period (8 April 2013–31 March 2023). The study was conducted at the Stockholm NSP, a harm reduction facility for PWID established in April 2013. In addition to distributing sterile needles, syringes, and other injecting equipment, the NSP provides counselling and wound care, conducts blood tests, gives out free HIV and hepatitis treatment, administers vaccinations for hepatitis A and B, and offers reproductive health services. The clinic also refers clients to somatic, psychiatric, or addiction care when needed. Amphetamine-type stimulants are the most commonly reported injected drugs among NSP clients, followed by opioids (mainly heroin). The use of highly potent substances, such as nitazenes or fentanyl, is rarely reported.

## The take-home naloxone program

The THN program at the Stockholm NSP, which provides naloxone free of charge, was launched in January 2018 and has been described in detail elsewhere [31]. The program, available to all NSP clients, provides brief training on recognising and responding to opioid overdoses using naloxone, along with information on risks of polydrug use and situations associated with reduced opioid tolerance. From January 2018 to March 2023, over 18,000 doses were distributed to 1,970 individual NSP clients. During this period, 2,323 unique overdoses when naloxone was administered were reported and documented.

## Study population

The study population included all individuals who visited the Stockholm NSP during the study period. Under Swedish law, individuals enrolling in an NSP are required to currently inject drugs, be at least 18 years old, and have a verified identity. The vast majority of individuals in Sweden have a unique personal identification number (PIN) assigned at birth or upon migration into the country. The PIN was used as the key identifier for data linkage between the two registers used in the study. Individuals without a Swedish PIN (*n* = 280) were excluded from the study due to the unavailability of register data on cause of death. Additionally, participants lacking baseline data from NSP enrolment (*n* = 24) were excluded.

## Data sources

### InfCare NSP

InfCare NSP is a national quality register used by the Stockholm NSP for routine data collection from all clients. It contains a wide range of data, including demographics, drug use, risk behaviours such as sharing of injection equipment, location of last injection (public or private setting), and sexual risk behaviours. The register also contains information on THN provision, vaccinations, blood test results, treatment for HIV and HCV, and information on participation in OAT. All data are collected during visits to the NSP and entered by clinical staff. While most data (e.g. demographics, drug use, and risk behaviour) are based on client self-report, other information, such as THN provision, vaccinations, and blood test results, is recorded based on clinical procedures and laboratory results. For the present analyses, key variables extracted from InfCare NSP included sex at birth, age at enrolment, housing situation, latest injected drug, injection frequency in the past month, HCV and HIV status, recent hospitalisation due to infection, ongoing contact with social services, psychiatry or addiction care, recent detoxification or rehabilitation, prison or custody, coerced care, and participation in OAT or THN programs. These variables were used to describe the cohort at baseline and as covariates in the regression analyses of all-cause and opioid overdose mortality.

Data are gathered at enrolment in the program and during regular follow-up interviews conducted every three to six months, with a more comprehensive questionnaire administered annually. Additional data are collected when naloxone refills are distributed as part of the THN program. At each visit participants report which drug they last injected. No active follow-up is carried out for clients who stop visiting the NSP.

### The Swedish National cause of death register

Pseudonymised register data on mortality and causes of death were provided by The Swedish National Board of Health and Welfare. The Swedish National Cause of Death Register (SNCDR) determines causes of death based on death certificates, covering more than 99% of deaths occurring in Sweden. The register provides a single underlying cause and, in relevant cases, contributing causes, using the English version of International Statistical Classification of Diseases and related Health Problems, 10th revision (ICD-10). The codes are recorded at the three- or four-character level, enabling classification by specific substance (e.g., T40.1 for heroin). Overall agreement between death certificates and causes derived from case summaries is high, though specificity can decline when moving from broader three-digit to more detailed four-digit codes [33]. SNCDR additionally contains data such as the date and place of death, and information on how the cause-of-death was ascertained (e.g. autopsy). The substance module, introduced in 2019, complements the SNCDR by recording specific substances involved in deaths caused by drug or medication poisonings (ICD codes X40-X44, X60-X64, Y10-Y14) [34].

### Data analysis

#### Outcomes

The primary outcome measure for this study was all-cause mortality. Secondary outcome measures included specific causes of death classified into four mutually exclusive categories: (1) opioid overdoses (see definition below); (2) external causes (other types of poisonings, violence, accidents, and suicides); (3) internal/natural causes (malignancy-related deaths, infection-related deaths, and other chronic disease-related deaths); and (4) other/unknown causes of death. All causes of death were determined using the underlying cause of death as provided by the SNCDR. A detailed overview of causes of death and ICD codes for the respective categories is provided in the supplementary material (See Table S1 and Table S2, Additional File 1).

In line with previous literature [35], opioid overdoses were defined as ICD-10 codes indicating unintentional or undetermined intentional poisoning (X40–X44, Y10–Y14) in the underlying cause of death, in combination with either an opioid-specific T code (T40.1-T40.4), or indication of an opioid in the SNCDR’s substance module. The decision to categorise overdoses with undetermined intent in the same group as accidental overdoses was based on a prior register-based study in Sweden, which found that poisonings classified as having undetermined intent more closely resembled accidental overdoses than suicides [36]. Nearly all deaths classified as opioid overdoses had undergone autopsy (99%).

### Key variables

We adjusted the regression models for baseline demographic variables including sex at birth (male/female) and age at NSP enrolment (divided into age groups 18–25, 26–35, 36–45, 46–55 and 56+), and time-varying variables, including housing situation categorised as (1) stable (i.e. own contract), (2) temporary (co-living, renting, couch-surfing, social service accommodation, hotels, trailer parks, etc.) or (3) homeless (sleeping on the street, in cars or in night shelters), type of drug used by injection categorised as: (1) amphetamine-type stimulants; (2) opioids; (3) opioids + other (opioids in combination with any other drug); or (4) other drugs. Time-varying variables also included injection frequency in the past month (daily/weekly/not at all), HIV status (negative/positive), hospitalisation due to infection in the past 12 months (yes/no), ongoing contact with social services, psychiatry or addiction care (yes/no), rehabilitation or detoxification in the past 12 months (yes/no), prison or custody in the past 12 months (yes/no). “Coerced care in the past 12 months” (yes/no) referred to any kind of involuntary care, such as compulsory care due to acute severe psychiatric illness or substance use. “Ever OAT” indicated whether the individual had participated in opioid agonist therapy at any point during the study period.

### Statistical analysis

We first investigated temporal trends in all-cause and cause-specific mortality using crude mortality rates (CMR) and standardised mortality ratios (SMR). We then examined predictors of all-cause and opioid overdose mortality using time-dependent regression models. To minimise misclassification and dilution of effects due to potential changes in time-varying variables such as type of drug injected or housing situation, we distinguished individuals who died within one year of their last recorded visit to the NSP from those who were alive or died later. We compared the baseline characteristics of participants based on their vital status at the study end date (alive, deceased, or deceased within one year of their last visit). Categorical variables were presented as counts and percentages, and continuous variables as both mean (standard deviation [SD]) and median (interquartile range [IQR]). Statistical tests were used to compare participants alive at study end with those who died during the study period. We used the χ² test for categorical variables and the Wilcoxon test for continuous variables.

CMR was calculated by summing all mortality events and dividing by the total person-time contributed during the study period. The result was expressed as deaths per 1,000 person-years (PY). SMR was used to compare the mortality of the cohort relative to the general Swedish population for years with full data (2014–2022). The number of deaths was related to the size of the population (average population) based on age- and sex-specific groups at the end of the year (i.e., age on 31 December). CMR and SMR were calculated for the entire study population, regardless of when during the study period participants died. Changes in SMR and CMR were tested using the Mann–Kendall test.

Person-time at risk was calculated from the date of the first visit until either death, end of study, or loss to follow-up, whichever occurred first. For the regression models, participants were censored and considered lost to follow-up if no revisit occurred within 365 days after their last visit. Consequently, these analyses focus on associations between mortality and participant characteristics among individuals who died within one year of their last recorded visit to the Stockholm NSP. To assess the robustness of our findings, we performed sensitivity analyses using alternative follow-up definitions (180 and 730 days) and applied inverse probability weighting (IPW) to account for potential informative censoring.

To analyse our primary outcome, all-cause mortality, we applied a time-dependent Cox regression, accounting for time-varying exposures and examining associations with potential predictors. Covariates for the univariable model were selected based on clinical relevance and prior international evidence from the literature on mortality among PWID. Multivariable analysis controlled for all covariates significant in univariable analysis (*p* < .05).

Furthermore, a time-dependent Fine and Gray regression model was used to examine the subdistribution hazards of opioid overdose deaths accounting for competing risks. The main event was defined as opioid overdose death and competing event was death from all other causes. The multivariable model controlled for sex, age, latest injected drug, injection frequency in the past month, ongoing contact with social services, psychiatry or addiction care, OAT status, and contact with rehabilitation, detoxification or coerced care in the past 12 months.

All presented p-values were two-sided, with significance set at *p* < .05. Missing values were rare: the highest proportion was for “latest injected drug” (2.7% of observations), while all other covariates had < 1% missing. For participants who returned but had had incomplete follow-up data, missing values were imputed using the last observed measurements from the same individual. All analyses and tabulations were performed using SAS 9.4 and R version 4.4.1. This study was not pre-registered and the results should be interpreted as exploratory.

## Results

### Study population

Out of 4,496 individuals who visited the Stockholm NSP during the study period, 280 were excluded due to the lack of a Swedish PIN, and 24 were excluded due to missing baseline data, resulting in a final sample of 4192 individuals eligible for inclusion. At baseline, the mean age of included participants was 37.6 years (SD ± 12.0). The majority were male (74%), and more than half (53%) reported amphetamine-type stimulants as their last injected drug. Most participants were either temporarily housed (44%) or homeless (18%) (Table 1).

**Table 1:** Baseline characteristics of Stockholm needle and syringe program (NSP) clients, 2013–2023 (*n* = 4192), overall and by vital status at study end (31 March 2023); deaths within 1 year of last NSP visit shown as a subset

	Total (*n* = 4192)	Alive (*n* = 3507)	Deceased (*n* = 685)	Deceased within 1 year from last visit (*n* = 388)
	*n* (%)	*n* (%)	*n* (%)	*n* (%)
Sex				
Female	1079 (26)	937 (27)	142 (21)	80 (21)
Male	3113 (74)	2570 (73)	543 (79)	308 (79)
Age at NSP enrolment (years)				
Mean (SD)	37.6 (± 12.0)	37.1 (± 11.7)	40.2 (± 12.8)	38.9 (± 12.7)
Median (IQR)	36.0 (28.0 to 47.0)	35.0 (27.0 to 46.0)	39.0 (29.0 to 50.0)	37.0 (28.0 to 49.0)
Age at first drug injection (years)				
Mean (SD)	22.7 (± 7.9)	22.8 (± 7.9)	22.0 (± 8.0)	22.2 (± 7.8)
Median (IQR)	20.0 (17.0 to 26.0)	21.0 (17.0 to 27.0)	20.0 (17.0 to 25.0)	20.0 (17.0 to 25.0)
Missing	150	132	18	9
Housing situation past 3 months				
Homeless	747 (18)	638 (18)	109 (16)	57 (15)
Temporary	1833 (44)	1510 (43)	323 (47)	197 (51)
Stable	1604 (38)	1352 (39)	252 (37)	134 (35)
Missing	8 (0)	7 (0)	1 (0)	–
Latest injected drug				
Amphetamine-type stimulants	2230 (53)	1923 (55)	307 (45)	166 (43)
Opioids	1840 (44)	1483 (42)	357 (52)	210 (54)
Opioids + other	34 (1)	27 (1)	7 (1)	5 (1)
Other	64 (2)	57 (2)	7 (1)	5 (1)
Missing	24 (1)	17 (0)	7 (1)	2 (1)
Injection frequency past month				
Daily	1984 (47)	1687 (48)	297 (43)	181 (47)
Weekly	1874 (45)	1554 (44)	320 (47)	172 (44)
Not at all	311 (7)	248 (7)	63 (9)	32 (8)
Missing	23 (1)	18 (1)	5 (1)	3 (1)
HCV status				
Exposed - active status	1621 (39)	1309 (37)	312 (46)	160 (41)
Exposed - cleared status	708 (17)	597 (17)	111 (16)	66 (17)
Exposed - unknown status	106 (3)	90 (3)	16 (2)	7 (2)
Never exposed	1266 (30)	1112 (32)	154 (22)	110 (28)
Missing	491 (12)	399 (11)	92 (13)	45 (12)
HIV status				
Negative	3588 (86)	3035 (87)	553 (81)	320 (82)
Positive	154 (4)	106 (3)	48 (7)	22 (6)
Missing	450 (11)	366 (10)	84 (12)	46 (12)
Hospitalisation due to infection past 12 months				
No	3721 (89)	3133 (89)	588 (86)	334 (86)
Yes	467 (11)	372 (11)	95 (14)	54 (14)
Missing	4 (0)	2 (0)	2 (0)	–
Ongoing contact with social services, psychiatry or addiction care				
No	689 (16)	633 (18)	56 (8)	37 (10)
Yes	3363 (80)	2774 (79)	589 (86)	333 (86)
Missing	140 (3)	100 (3)	40 (6)	18 (5)
Ever OAT*				
No	3075 (73)	2579 (74)	496 (72)	294 (76)
Yes	1117 (27)	928 (26)	189 (28)	94 (24)
Rehabilitation past 12 months				
No	3335 (80)	2818 (80)	517 (75)	300 (77)
Yes	825 (20)	665 (19)	160 (23)	84 (22)
Missing	32 (1)	24 (1)	8 (1)	4 (1)
Detoxification past 12 months				
No	2924 (70)	2477 (71)	447 (65)	245 (63)
Yes	1229 (29)	1000 (29)	229 (33)	138 (36)
Missing	39 (1)	30 (1)	9 (1)	5 (1)
Prison or custody past 12 months				
No	3267 (78)	2725 (78)	542 (79)	301 (78)
Yes	921 (22)	780 (22)	141 (21)	87 (22)
Missing	4 (0)	2 (0)	2 (0)	–
Coerced care past 12 months				
No	3791 (90)	3174 (91)	617 (90)	350 (90)
Yes	397 (9)	331 (9)	66 (10)	38 (10)
Missing	4 (0)	2 (0)	2 (0)	–

SD = standard deviation; IQR = interquartile range; NSP = Needle and Syringe Program; HCV = Hepatitis C virus; HIV = Human immunodeficiency virus; OAT = Opioid agonist therapy

*Ever OAT denotes any episode of opioid agonist therapy during the study period (2013–2023)

### Primary outcome

Over the ten-year study period, 685 individuals (16.3%) died. Of these, 388 (57%) died within one year of their last visit to the Stockholm NSP (Table 1). Several characteristics differed between participants based on their vital status at study end. Compared to those who survived, deceased individuals were more likely to be male (*p* < .01), older at enrolment (*p* < .01), younger at first drug injection (*p* < .01), HIV positive (*p* < .01), have an active HCV infection (*p* < .01), and to have ongoing contact with social services, psychiatry, or addiction care (*p* < .01). Participants who died were also more likely to have injected opioids (*p* < .01), to have been hospitalised due to an infection in the past 12 months (*p* = .01), to have injected only weekly or not at all in the past month (*p* = .03), and to have spent time in detoxification (*p* = .01) or rehabilitation (*p* = .01) in the past 12 months (Table 1).

### All-cause mortality

**Fig. 1:**
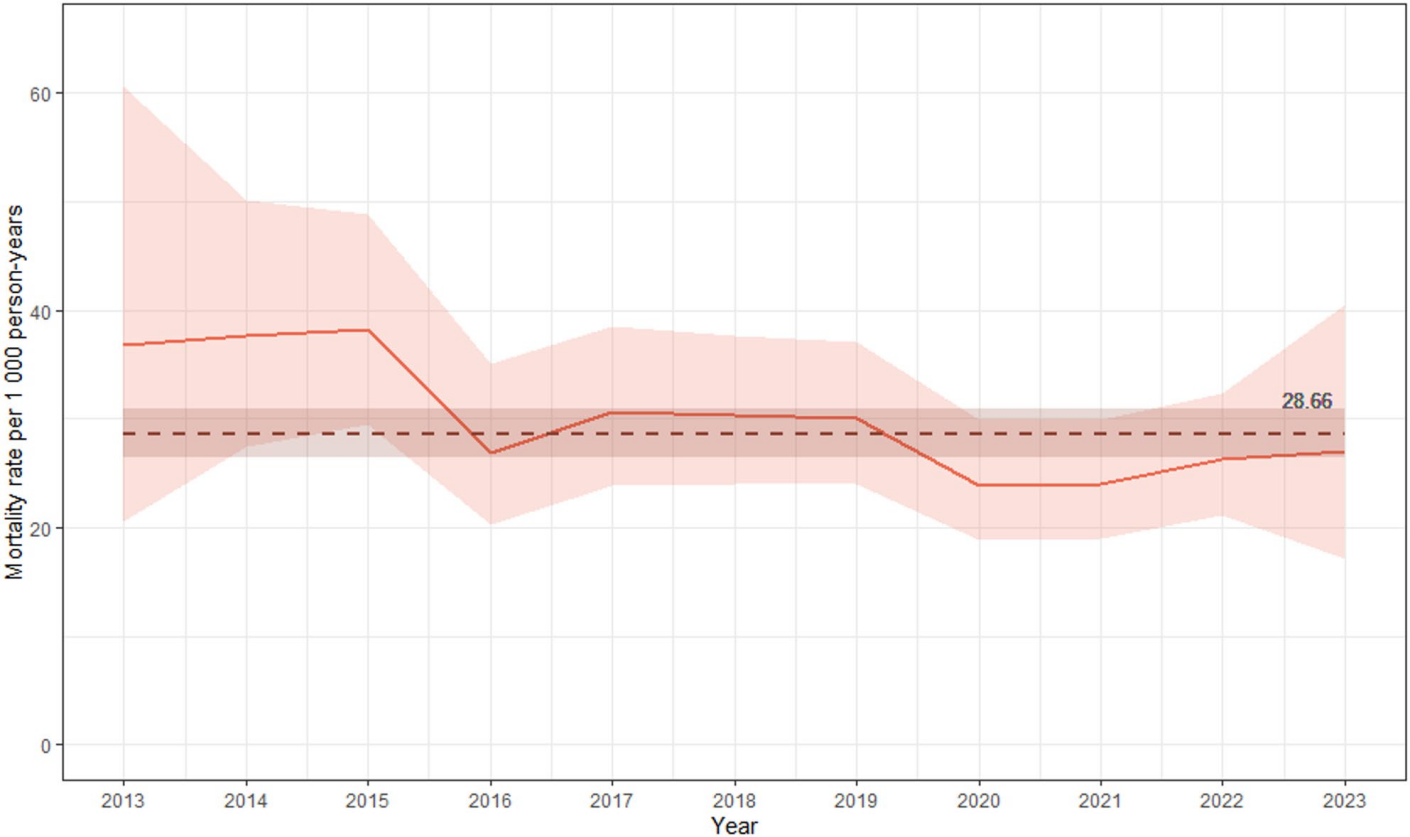
All-cause crude mortality rate (CMR) per 1000 person-years among Stockholm Needle and Syringe Program (NSP) clients 2013–2023 (*n* = 4192). Total period CMR is shown as a dashed horizontal line (28.66; 95% confidence interval: 26.41, 30.86). The downward trend is statistically significant (Mann-Kendall test *p* = .03)

Over the study period, the observed all-cause CMR showed a significant decline from 36.75 per 1,000 PY in 2013 to 27.04 in 2023 (*p* = .03), with a mean value of 28.66 (CI 26.41, 30.86) per 1,000 PY over the entire period (Fig. 1).

**Fig. 2:**
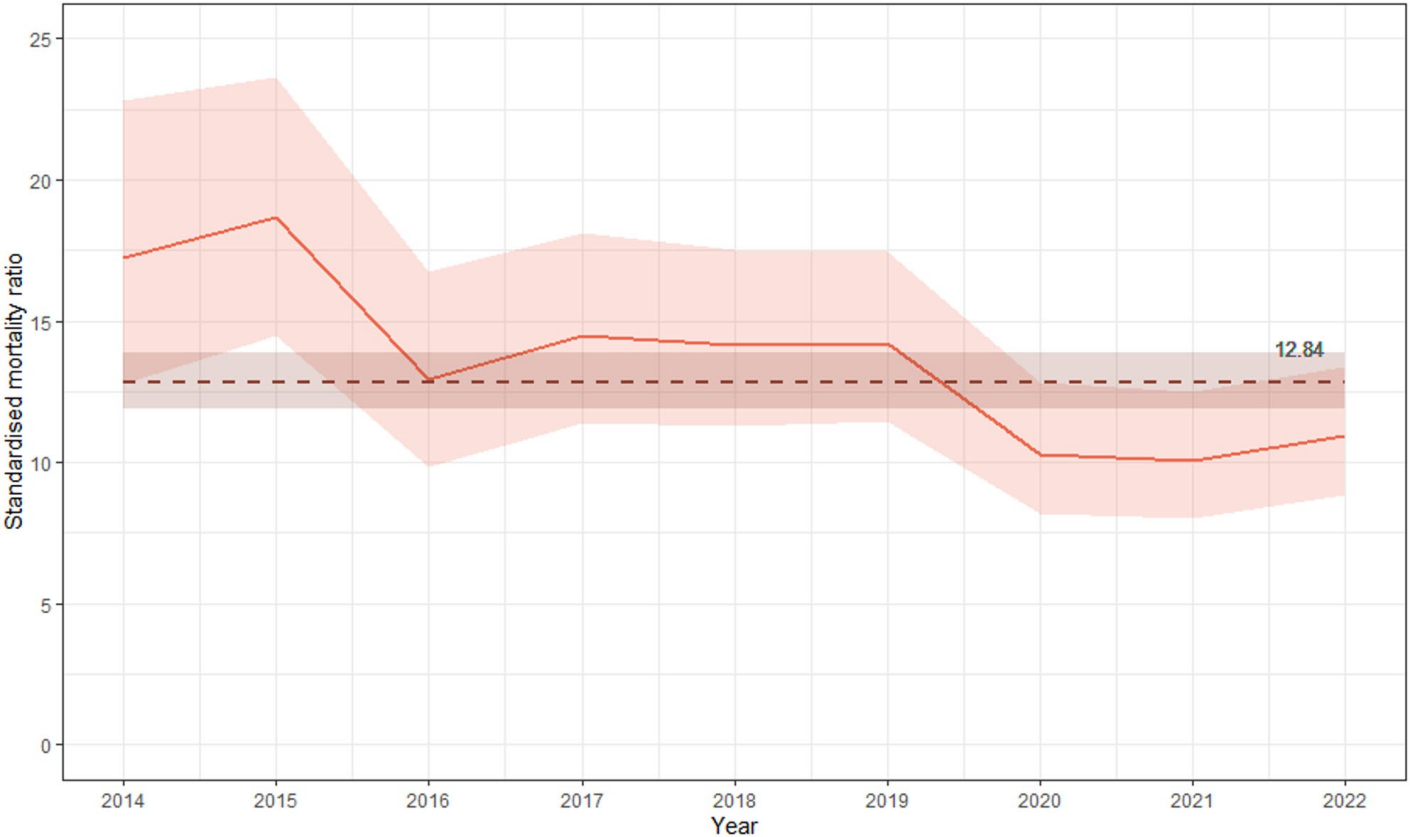
All-cause age- and sex-standardised mortality ratio (SMR) among Stockholm Needle and Syringe Program (NSP) clients 2014–2022 (*n* = 4192). Total period SMR is shown as a dashed horizontal line (12.84; 95% confidence interval: 11.88, 13.86). The downward trend is statistically significant (Mann-Kendall test *p* = .03)

In 2014 (the first year with full data), mortality was 17.24 times higher than in the age- and sex-matched general population. However, the SMR declined significantly over time, falling to 10.94 in 2022 (*p* = .03), the final year with full data. The mean all-cause SMR across 2014–2022 was 12.84 (Fig. 2).

### Characteristics and causes of deaths

The mean age at death was 43.6 years (SD ± 13.5), with a range from 38.4 years (SD ± 11.3) for those who died from opioid overdose to 55.7 years (SD ± 10.8) for those who died from internal/natural causes (Table 2). The most common location of death was at a private residence (39%), with nearly half (48%) of all opioid overdose deaths occurring in this setting. The proportion of deaths occurring within one year of the last NSP visit varied by cause, ranging from 38% for internal/natural causes to 65% for opioid overdose deaths (Table 2, last row).

**Table 2:** Characteristics of all-cause and cause-specific mortality among Stockholm needle and syringe program (NSP) clients 2013–2023 (*n* = 685 decedents).

	All-cause deaths (n = 685)	Opioid overdose deaths n = 363 (53%)	External causes n = 136 (20%)	Internal/natural causes n = 154 (22%)	Other/unknown causes n = 32 (5%)
	n (%)	n (%)	n (%)	n (%)	n (%)
Sex					
Female	142 (21)	79 (22)	25 (18)	33 (21)	5 (16)
Male	543 (79)	284 (78)	111 (82)	121 (79)	27 (84)
Age at NSP enrolment (years)					
Mean (SD)	40.2 (± 12.8)	35.4 (± 10.8)	38.7 (± 12.1)	51.3 (± 10.5)	47.0 (± 12.9)
Median (IQR)	39.0 (29.0 to 50.0)	34.0 (27.0 to 42.0)	38.0 (28.0 to 48.0)	52.5 (46.0–58.0)	51.0 (34.0 to 55.5)
Age at death (years)					
Mean (SD)	43.6 (± 13.5)	38.4 (± 11.3)	42.2 (± 12.4)	55.7 (± 10.8)	50.7 (± 12.6)
Median (IQR)	43.0 (32.0 to 54.0)	37.0 (29.0 to 46.0)	41.0 (32.0 to 51.5)	57 (49–63.3)	54.5 (37.0 to 59.5)
Housing situation past 3 months					
Homeless	109 (16)	58 (16)	23 (17)	24 (16)	4 (13)
Temporary	323 (47)	182 (50)	62 (46)	67 (44)	12 (38)
Stable	252 (37)	122 (34)	51 (38)	63 (41)	16 (50)
Missing	1 (0)	1 (0)	–	–	–
Latest injected drug					
Amphetamine-type stimulants	307 (45)	114 (31)	78 (57)	98 (64)	17 (53)
Opioids	357 (52)	237 (65)	53 (39)	52 (34)	15 (47)
Opioids + other	7 (1)	3 (1)	3 (2)	1 (0)	–
Other	7 (1)	5 (1)	1 (1)	1 (0)	–
Missing	7 (1)	4 (1)	1 (1)	2 (1)	–
Injection frequency past month					
Daily	297 (43)	155 (43)	61 (45)	69 (45)	12 (38)
Weekly	320 (47)	169 (47)	64 (47)	69 (45)	18 (56)
Not at all	63 (9)	38 (10)	9 (7)	14 (9)	2 (6)
Missing	5 (1)	1 (0)	2 (1)	2 (1)	–
HCV status					
Exposed—active status	312 (46)	146 (40)	67 (49)	83 (54)	16 (50)
Exposed—cleared status	111 (16)	58 (16)	19 (14)	30 (19)	4 (13)
Exposed—unknown status	16 (2)	10 (3)	–	5 (3)	1 (3)
Never exposed	154 (22)	111 (31)	29 (21)	10 (6)	4 (13)
Missing	92 (13)	38 (10)	21 (15)	26 (17)	7 (22)
HIV status					
Negative	553 (81)	311 (86)	108 (79)	111 (72)	23 (72)
Positive	48 (7)	16 (4)	7 (5)	22 (14)	3 (9)
Missing	84 (12)	36 (10)	21 (15)	21 (14)	6 (19)
Hospitalisation due to infection past 12 months					
No	588 (86)	320 (88)	108 (79)	131 (85)	29 (91)
Yes	95 (14)	42 (12)	27 (20)	23 (15)	3 (9)
Missing	2 (0)	1 (0)	1 (1)	-	-
Ongoing contact with social services, psychiatry or addiction care					
No	56 (8)	22 (6)	16 (12)	14 (9)	4 (13)
Yes	589 (86)	319 (88)	114 (84)	129 (84)	27 (84)
Missing	40 (6)	22 (6)	6 (4)	11 (7)	1 (3)
Rehabilitation past 12 months					
No	517 (75)	259 (71)	98 (72)	134 (87)	26 (81)
Yes	160 (23)	99 (27)	36 (26)	19 (12)	6 (19)
Missing	8 (1)	5 (1)	2 (1)	1 (0)	–
Detoxification past 12 months					
No	447 (65)	212 (58)	82 (60)	127 (82)	26 (81)
Yes	229 (33)	148 (41)	49 (36)	26 (17)	6 (19)
Missing	9 (1)	3 (1)	5 (4)	1 (0)	–
Prison or custody past 12 months					
No	542 (79)	295 (81)	99 (73)	126 (82)	22 (69)
Yes	141 (21)	67 (18)	36 (26)	28 (18)	10 (31)
Missing	2 (0)	1 (0)	1 (1)	–	–
Coerced care past 12 months					
No	617 (90)	318 (88)	121 (89)	150 (97)	28 (88)
Yes	66 (10)	44 (12)	14 (10)	4 (3)	4 (13)
Missing	2 (0)	1 (0)	1 (1)	–	–
Location of death					
Care home/hospital	229 (33)	79 (22)	38 (28)	105 (68)	7 (22)
Private accommodation	264 (39)	174 (48)	41 (30)	36 (23)	13 (41)
Other/unknown	175 (26)	106 (29)	56 (41)	11 (7)	2 (6)
Missing	17 (2)	4 (1)	1 (1)	2 (1)	10 (31)
Deceased < 1 year after last NSP visit					
No	297 (43)	126 (35)	58 (43)	95 (62)	18 (56)
Yes	388 (57)	237 (65)	78 (57)	59 (38)	14 (44)

SD = standard deviation; IQR = interquartile range; NSP = Needle and Syringe Program; HCV = Hepatitis C virus; HIV = Human immunodeficiency virus.

The analysis of cause-specific mortality revealed that opioid overdose was the leading cause of death, accounting for 53% of all deaths throughout the study period. Internal/natural causes were the second most common, responsible for 22% of deaths, followed by external causes, which accounted for 20% (Table 2). Detailed breakdowns of cause-specific deaths are available in the additional file (see Table S1, Additional File 1).

### Temporal trends in cause-specific mortality

**Fig. 3:**
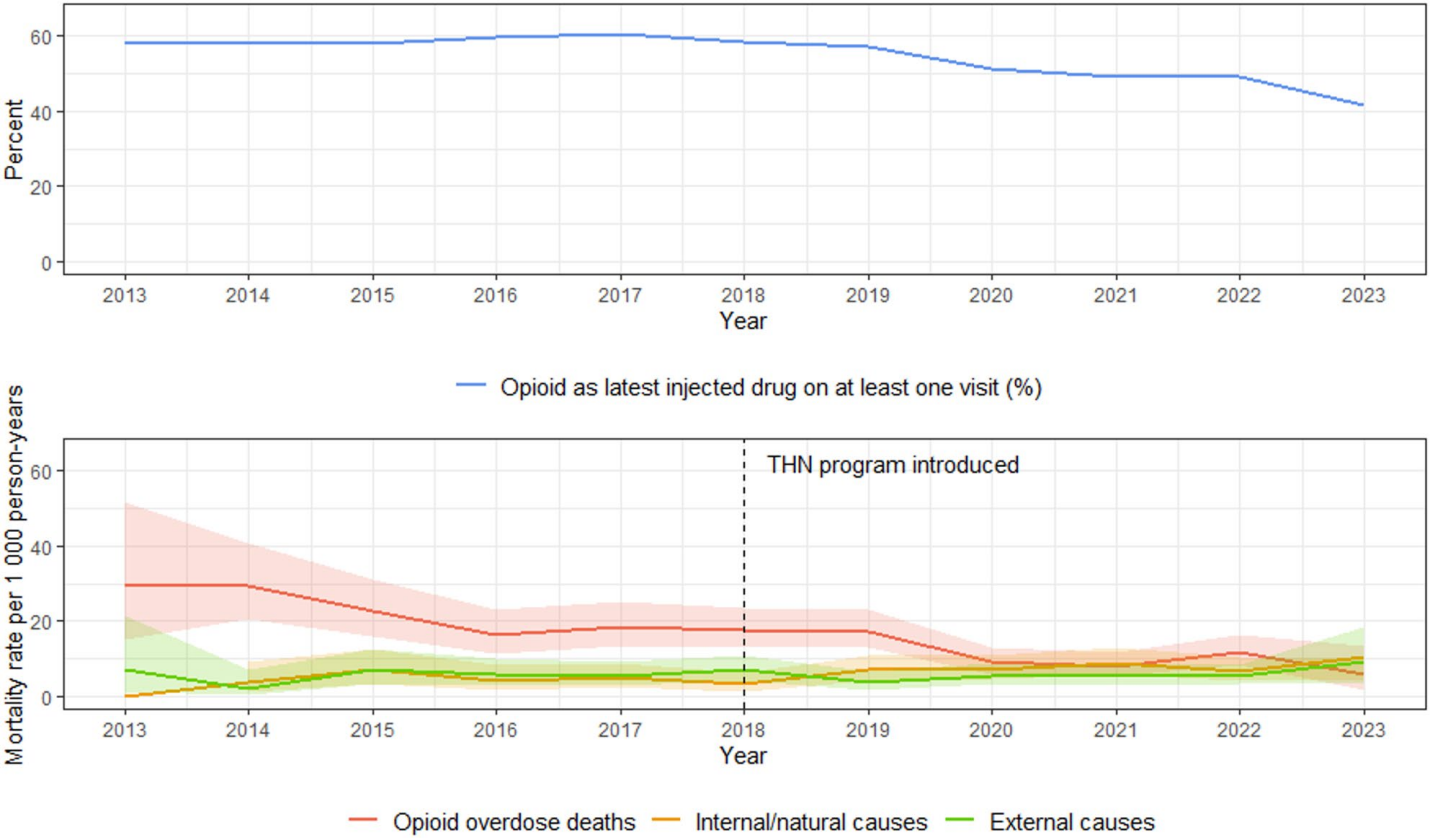
Cause-specific crude mortality rate (CMR) per 1,000 person-years and reported opioid use (%) among Stockholm Needle and Syringe Program (NSP) clients 2013–2023 (*n* = 4192). Opioid use was defined as opioids recorded as the latest injected drug at ≥1 visit within the calendar year. The vertical dashed line marks implementation of Take-HomeNaloxone (THN) at the Stockholm NSP (January 2018). The downward trend in opioid overdose mortality and the upward trend in internal/natural-cause mortality were statistically significant (Mann-Kendall test: p < 0.01 and p = .01, respectively), whereas no significant trend was detected for external causes (p = 1.00). The 95% confidence interval for internal/natural causes in 2013 is not shown because no deaths occurred in that category that year

In addition to all-cause mortality, we examined temporal trends in cause-specific mortality. Over the study period, the mortality rate attributed to opioid overdose declined substantially from 29.40 per 1,000 PY in 2013 to 5.88 per 1,000 PY in 2023 (*p* < .01). This decrease coincided with a decline in the annual proportion of NSP participants who ever reported injecting opioids, from 58% in 2013 to 41% in 2023. While opioid overdose deaths decreased, mortality rates due to internal/natural causes increased during the same period (*p* = .01), whereas deaths from external causes showed no significant change (*p* = 1.00) (Fig. 3).

### Predictors of all-cause and opioid overdose mortality

After investigating temporal trends in all-cause and cause-specific mortality, we used time-dependent regression models to examine predictors of all-cause and opioid overdose mortality (Table 3). Since opioid overdoses constituted 53% of deaths, several predictors overlapped with those for all-cause mortality. Opioids as the latest injected drug was the dominant predictor of overdose death (sHR 4.20; CI 2.94, 6.00) and was also associated with higher all-cause mortality (aHR 2.63; CI 2.07, 3.34). Non-daily recent injecting and recent detoxification were linked to increased risk in both multivariable models, with larger effects for opioid overdose death. Ongoing contact with social services, psychiatry or addiction care was associated with higher risk across both outcomes. In contrast, male sex and older age at NSP enrolment (56 + years) were associated with higher risk of all-cause mortality, but not independently with opioid overdose death. OAT participation was associated with a strongly protective effect for both all-cause mortality (aHR 0.30; CI 0.23, 0.40) and opioid overdose death (sHR 0.31; CI 0.22, 0.42). Sensitivity analyses, including alternative follow-up periods (180 or 730 days) and inverse probability weighting for censoring, confirmed the robustness of the main findings.

**Table 3:** Univariable and multivariable regression analyses of all-cause mortality and overdose deaths among Stockholm needle and syringe program (NSP) clients 2013–2023 (*n* = 4192)

	All-cause mortality		Opioid overdose death	
	Univariable analysis	Multivariable analysis	Univariable analysis	Multivariable analysis
	HR (95% CI)	aHR (95% CI)	sHR (95% CI)	sHR (95% CI)
Sex				
Female (reference)	1	1	1	1
Male	**1.37 (1.08, 1.76)**	**1.34 (1.04, 1.74)**	1.27 (0.94, 1.73)	1.25 (0.90, 1.72)
Age at NSP enrolment				
18–25 (reference)	1	1	1	1
26–35	0.81 (0.60, 1.10)	0.96 (0.70, 1.31)	0.78 (0.55, 1.10)	0.97 (0.67, 1.39)
36–45	0.78 (0.57, 1.08)	1.00 (0.71, 1.41)	0.70 (0.48, 1.01)	0.97 (0.64, 1.46)
46–55	0.87 (0.63, 1.20)	1.21 (0.85, 1.71)	**0.51 (0.34, 0.78)**	0.85 (0.51, 1.40)
56 +	1.15 (0.79, 1.69)	**1.82 (1.20, 2.76**)	**0.20 (0.09, 0.47)**	0.41 (0.17, 1.01)
Housing situation past 3 months				
Homeless (reference)	1	–	1	–
Temporary	1.29 (0.97, 1.70)	–	1.22 (0.86, 1.72)	–
Stable	0.99 (0.74, 1.34)	–	0.93 (0.64, 1.35)	–
Latest injected drug				
Amphetamine-type stimulants (reference)	1	1	1	1
Opioid or opioids + other	**1.81 (1.48, 2.23)**	**2.63 (2.07, 3.34)**	**3.57 (2.66, 4.79)**	**4.20 (2.94, 6.00)**
Injection frequency past month				
Daily (reference)	1	1	1	1
Weekly	**1.32 (1.07, 1.64)**	**1.39 (1.11, 1.74)**	**1.33 (1.01, 1.76)**	**1.58 (1.18, 2.12)**
Not at all	**1.46 (1.04, 2.05)**	1.43 (0.98, 2.06)	**1.79 (1.18, 2.71)**	**1.98 (1.26, 3.12)**
HIV status				
Negative (reference)	1	–	–	–
Positive	1.10 (0.72, 1.66)	–	–	–
Hospitalisation due to infection past 12 months				
No (reference)	1	–	–	–
Yes	1.27 (0.96, 1.68)	–	–	–
Ongoing contact with social services, psychiatry or addiction care				
No (reference)	1	1	1	1
Yes	**1.53 (1.12, 2.09)**	**1.78 (1.28, 2.46)**	**1.89 (1.22, 2.91)**	**1.80 (1.15, 2.82)**
Ever OAT				
No (reference)	1	1	1	1
Yes	**0.57 (0.45, 0.72)**	**0.30 (0.23, 0.40)**	**0.68 (0.52, 0.89)**	**0.31 (0.22, 0.42)**
Rehabilitation past 12 months				
No (reference)	1	–	1	1
Yes	1.21 (0.95, 1.55)	–	**1.40 (1.04, 1.89)**	0.88 (0.63, 1.23)
Detoxification past 12 months				
No (reference)	1	1	1	1
Yes	**1.46 (1.19, 1.80)**	**1.56 (1.25, 1.95)**	**2.03 (1.57, 2.62)**	**1.77 (1.31, 2.39)**
Prison or custody past 12 months				
No (reference)	1	–	1	–
Yes	0.98 (0.78, 1.24)	–	0.95 (0.70, 1.29)	–
Coerced care past 12 months				
No (reference)	1	–	1	1
Yes	1.20 (0.86, 1.68)	–	**1.64 (1.13, 2.39)**	1.12 (0.74, 1.69)

Bold type indicates statistically significant results (*p* < .05). HR = hazard ratio; aHR = adjusted hazard ratio; CI = confidence interval; sHR = subdistribution hazard ratio; NSP = Needle and Syringe Program; HIV = Human immunodeficiency virus; OAT = Opioid agonist therapy

HR and aHR for all-cause mortality were estimated using Cox time-dependent regression model

sHR for opioid overdose deaths were estimated based on Fine and Gray’s time-dependent regression model, accounting for competing risks

## Discussion

In this study, we investigated the evolving risk of mortality and causes of death in a large cohort of Swedish NSP clients during a ten-year period. Our findings highlight the significant burden of mortality in this population, with more than 16% of the total cohort having died during the study. Importantly, over half of the deaths occurred within one year of the last NSP visit, underscoring the acute risks faced by PWID with current or recent injecting drug use.

Mortality in this population of PWID substantially exceeded that of the Swedish general population with a mean SMR of 12.84, which further emphasises the disproportionate mortality burden experienced by PWID. This is slightly higher than estimates reported in other PWID cohorts, where SMRs ranged from 8.3 to 12.4 [7, 16, 37–39], yet lower than figures from Greece and Australia, which found SMRs of 15.86 and 16.64, respectively [40, 41]. While the study cohort predominantly reported using amphetamine-type stimulants, which has been associated with lower mortality [42], the observed CMR of 28.66 per 1,000 person-years is consistent with a recent meta-analysis that reported a pooled all-cause CMR of 27.1 per 1,000 person-years among PWID who use opioids [43].

When interpreting predictors of all-cause mortality, it is important to consider that opioid overdoses accounted for more than half of all deaths in our data. Consequently, many risk factors for overdose, such as opioid use, non-daily injecting, and recent detoxification, also drive all-cause mortality, with generally stronger associations for overdose. Ongoing contact with social services, psychiatry or addiction care was associated with higher risk across outcomes, likely reflecting greater underlying severity and help-seeking patterns during periods of instability. In contrast, male sex and older age at enrolment were associated with all-cause mortality but were not independent predictors of overdose death. These patterns align with prior research showing increased all-cause mortality with older age among PWID [7, 41, 44]. While the association between ageing and mortality is expected, it is particularly relevant among people who use drugs, where advancing age often coincides with an accumulation of comorbidities, prolonged exposure to drug-related harms, and long-term complications related to drug use [45].

In our cohort, infrequent injecting was most strongly associated with overdose mortality and similar associations with all-cause mortality have been observed in other contexts [46, 47]. This association may reflect periods without injecting due to time spent in institutional settings such as prisons, rehabilitation centres, or coerced care [12, 13], or intentional pauses or reductions in drug use (e.g. personal choice), both of which reduce tolerance and elevate overdose risk when injecting drug use is resumed. This subgroup may also include individuals who use drugs sporadically due to declining health or chronic illnesses, which could explain their increased risk of mortality [47].

In our analysis of cause-specific deaths, we found that while opioid overdose deaths declined over time, deaths from internal/natural causes increased. This shift may reflect changing health-related vulnerabilities among the Stockholm NSP clients, suggesting a direction for the continued development of the Stockholm NSP as a comprehensive low-threshold clinic, addressing both drug-related risks and the broader health needs of PWID [48]. However, we did not investigate whether the age distribution of the population at risk changed during the study period. It is possible that the cohort aged over time, contributing to a higher baseline risk for chronic diseases and natural causes of death [4]. Future research could explore age-related trends and comorbidities in the Stockholm NSP population to better understand the observed increase in mortality from internal/natural causes.

The observed decline in opioid overdose deaths occurred over a period that included the introduction of THN at the Stockholm NSP in 2018. This reduction is encouraging and aligns with previous studies that have documented reductions in opioid overdose mortality following the implementation of THN programs in Sweden [32] as well as internationally [35, 49]. However, the social nature of overdose prevention with THN also highlights critical gaps in existing harm reduction strategies. Although substantial, the share of opioid overdose deaths in private residences in our cohort (48%) was lower than the 81% reported in a previous Swedish study of opioid-related fatalities [50]. Moreover, we lack information on whether bystanders were present at the time of death, making it difficult to assess whether naloxone access could have reduced mortality. Supervised consumption sites (SCS), where individuals can use drugs under medical supervision, are not available in Sweden. A recent study on willingness to use SCS among Stockholm NSP clients suggested that such facilities could appeal to both individuals who typically use drugs in public and those who use them at home but lack a safety net [51]. Expanding harm reduction strategies to include SCS may offer an additional way to prevent overdose deaths among PWID in Stockholm, particularly for those who use drugs alone or in unprotected environments.

The downward trend in opioid overdose deaths must also be interpreted in the broader context of concurrent changes in drug use patterns and harm reduction efforts. Our analysis indicates a decline in reported opioid use among NSP clients over time, which inherently reduces the risk of opioid overdose. Over the study period, access to OAT in the Stockholm region expanded, particularly following a revision of national guidelines in 2016 that broadened eligibility criteria [52]. This policy shift may have contributed to an increase in OAT coverage at the population level, which could have played a role in reducing overdose mortality in our study cohort. Consistent with this, individuals who had ever received OAT during the study period had a significantly lower risk of opioid overdose death. Although our OAT variable did not account for treatment duration or changes over time, this finding aligns with previous research highlighting the protective effects of OAT [24]. The COVID-19 pandemic also overlapped with parts of the study period, which affected access to heroin and may have further influenced mortality rates [53]. While the introduction of THN may have contributed to the decline in overdose deaths, disentangling its specific impact from these concurrent factors remains challenging.

This study has several limitations that should be considered when interpreting its findings. The cohort comprised clients from an NSP, representing a community-based, retrospective cohort. Consequently, inherent limitations associated with this study design apply, including potential selection and information biases. Although the underlying causes of death were extracted from a national mortality register of high quality, some degree of misclassification in cause-of-death coding may still remain, particularly at detailed ICD-10 subcodes. Substance-specific results should therefore be interpreted with caution.

NSP participation was used as a proxy for active injecting drug use, however we could not determine the drug use status of individuals who discontinued NSP visits. In several cases, participants died years after their last recorded visit, during which time there was a lack of updated information on time-dependent covariates. To address this limitation, we censored participants who had not returned to the NSP within 365 days, aiming to reduce misclassification of time at risk.

Furthermore, we acknowledge that OAT participation was coded as ever receiving OAT during the study period. This cumulative coding risks immortal-time bias, as person-time prior to OAT initiation may be misclassified as exposed, potentially biasing associations in favour of a protective effect.

Additionally, there are a number of coexisting risk factors for premature death that our study was not able to account for. While we had access to extensive data on demography, drug use, risk behaviour and socio-economic factors, we were not able to control for all potential health related confounders. Lastly, we had to exclude people who lacked a Swedish PIN, which may underestimate mortality rates and further limit the generalisability of our findings.

Despite these limitations, the study also has several notable strengths. Conducted in a setting with a high burden of drug-related mortality, we were able to quantify long-term mortality trends over a ten-year period among more than 4,000 PWID—a population that is often considered to be hard to reach. Linkage to a comprehensive national mortality register, with high coverage and death ascertainment, allowed for robust and reliable mortality estimates. Moreover, the infrastructure of the Stockholm NSP facilitated the collection of detailed, individual-level data, enabling an in-depth analysis of systematically recorded cause-of-death information.

## Conclusions

All-cause mortality declined over the study period, alongside pronounced reductions in opioid overdose deaths, coinciding with expanded harm-reduction services and a decline in reported opioid injection among study participants.

Our results indicate promising effects of current harm reduction efforts and highlight the continued need to expand access to OAT, increase THN distribution, and consider the implementation of SCS to further address mortality among PWID in Sweden.

## Supplementary Information

Below is the link to the electronic supplementary material.

Supplementary Material 1

## Data Availability

The data used in this study are not publicly available, as the ethical approval explicitly states that the data will not be shared in order to protect participant confidentiality.

## Abbreviations

AHR: Adjusted hazard ratio
CI: Confidence interval
CMR: Crude mortality rate
COVID-19: Coronavirus disease 2019
HCV: Hepatitis C virus
HIV: Human immunodeficiency virus
HR: Hazard ratio
ICD-10: International Statistical Classification of Diseases and related Health Problems
InfCare NSP: InfCare Needle and syringe program
IPW: Inverse probability weighting
IQR: Interquartile range
NSP: Needle and syringe program
OAT: Opioid agonist therapy
PIN: Personal identification number
PWID: People who inject drugs
PY: Person-years
SCS: Supervised consumption sites
SD: Standard deviation
SMR: Standardised mortality ratio
SNCDR: The Swedish National Cause of Death Register
SHR: Subdistribution hazard ratio
THN: Take-Home Naloxone
