# Supplementary material for: All-cause mortality and overdose deaths among 4192 people who inject drugs in Stockholm: a 10-year register-based cohort study
Source: Harm Reduct J. 2026 Feb 9;23:41. doi: 10.1186/s12954-026-01407-z (PMC12934103; doi:10.1186/s12954-026-01407-z)
Supplement: Supplementary file 1 — Supplementary Material 1 [file 12954_2026_1407_MOESM1_ESM.docx]

**Additional File 1**

Table S1. Distribution of causes of death among Stockholm Needle and Syringe Program (NSP) clients 2013–2023 (n = 685 decedents)

| **Death Group** | **Cause of death** | **N** | **% within category** | **% of total** |
| --- | --- | --- | --- | --- |
| **Opioid overdoses (n = 363)** | Accidental opioid overdose | 284 | 78.2 | 41.5 |
|  | Opioid overdose with undetermined intent | 79 | 21.8 | 11.5 |
| **External causes (n = 136)** | Other poisoning | 50 | 36.8 | 7.3 |
|  | Accident and adverse events | 36 | 26.5 | 5.3 |
|  | Assault | 5 | 3.7 | 0.7 |
|  | Suicide | 45 | 33.1 | 6.5 |
| **Internal/natural causes (n = 154)** | HIV infection | 3 | 1.9 | 0.4 |
|  | Other infection | 15 | 9.7 | 2.2 |
|  | Septicaemia | 5 | 3.2 | 0.7 |
|  | Pneumonia and influenza | 8 | 5.2 | 1.2 |
|  | Malignancy | 44 | 28.6 | 6.4 |
|  | Heart disease | 43 | 27.9 | 6.3 |
|  | Liver disease | 12 | 7.8 | 1.8 |
|  | Diabetes | 6 | 3.9 | 0.9 |
|  | Renal | 3 | 1.9 | 0.4 |
|  | Chronic lung disease | 9 | 5.8 | 1.3 |
|  | Other chronic | 2 | 1.3 | 0.3 |
|  | Cerebrovascular | 4 | 2.6 | 0.6 |
| **Other/miscellaneous/unknown causes (n = 32)** | Miscellaneous | 6 | 18.8 | 0.9 |
|  | Haemorrhage | 8 | 25 | 1.2 |
|  | Unknown cause | 18 | 56.3 | 2.6 |

Table S2. ICD-10 codes for underlying causes of death by cause-of-death group among Stockholm Needle and Syringe Program (NSP) clients 2013–2023

| **Death Group** | **Cause of death** | **ICD-10 codes** |
| --- | --- | --- |
| **Opioid overdoses** | Accidental opioid overdoses | X41, X42, X44 ***** |
|  | Opioid overdoses with undetermined intent | Y11, Y12, Y14 ***** |
| **External causes** | Other poisoning | F102, F191, F192, Y15, X41, X42, X44, Y11, Y12, Y14 |
|  | Accidents and adverse events | V051, V234, V274, V435, V436, V485, W10, W17, W18, W19, W23, W69, W74, W79, X00, X08, X31, X47, X599, Y20, Y21,Y29, Y31, Y33, Y34,  Y86 |
|  | Assault | X99, Y00 |
|  | Suicide | X60, X61, X62, X64  X70, X71, X78, X80, X81, X670 |
| **Internal/natural causes** | HIV infection | B208, B24 |
|  | Other infection | M009, B376, B99, B182, G039, I301, I330, I339 |
|  | Septicaemia | A409, A412, A419 |
|  | Pneumonia and influenza | J851, U071, J189, J209 |
|  | Malignancy | C049, C61, C159, C163, C169, C179, C187, C220, C229, C259, C269, C329, C349, C435, C539, C800, C809, C851, C859, C900, C920 |
|  | Heart disease | I080, I213, I219, I249, I251, I258, I259, I269, I272, I409, I420, I426, I509, I514, I517, I519, Q256 |
|  | Liver disease | K703, K729, K746, K861, K859 |
|  | Diabetes | E100, E101, E110, E115, E140, E141 |
|  | Renal | N184, N185, N189 |
|  | Chronic lung disease | J82, J440, J449, J961, J981 |
|  | Other chronic | G309, K254 |
|  | Cerebrovascular | I633, I639, I679, I693 |
| **Other/miscellaneous/unknown causes** | Miscellaneous | I850, I710, K529, K566, K622, |
|  | Haemorrhage | I601, I609, I619, I620 |
|  | Unknown causes | R990, R998, R999 |

**=in combination with either a contributing T-code indicating opioids (T40.0, T40.1, T40.2, T40.3 or T40.4) or that opioids were marked in the substance module. HIV= Human immunodeficiency virus.*

*ICD-10= International Statistical Classification of Diseases and related Health Problems, 10^th^ revision. This table includes only ICD-10 codes that were recorded as the underlying cause of death in our dataset. Each deceased individual is represented by a single ICD code.*
